# Tooth Type Enhanced Transformer for Children Caries Diagnosis on Dental Panoramic Radiographs

**DOI:** 10.3390/diagnostics13040689

**Published:** 2023-02-12

**Authors:** Xiaojie Zhou, Guoxia Yu, Qiyue Yin, Jun Yang, Jiangyang Sun, Shengyi Lv, Qing Shi

**Affiliations:** 1 Department of Stomatology, Beijing Children’s Hospital, Capital Medical University, National Center for Children’s Health, Beijing 100045, China; 2 Department of Stomatology, National Clinical Research Center for Respiratory Diseases, Beijing Children’s Hospital, Capital Medical University, National Center for Children’s Health, Beijing 100045, China; 3 Institute of Automation, Chinese Academy of Sciences, Beijing 100190, China; 4 Department of Automation, Tsinghua University, Beijing 100084, China; 5 Beijing Stomatological Hospital, Capital Medical University, Beijing 100050, China

**Keywords:** caries diagnosis, transformer, dental panoramic radiographs, children, artificial intelligence

## Abstract

The objective of this study was to introduce a novel deep learning technique for more accurate children caries diagnosis on dental panoramic radiographs. Specifically, a swin transformer is introduced, which is compared with the state-of-the-art convolutional neural network (CNN) methods that are widely used for caries diagnosis. A tooth type enhanced swin transformer is further proposed by considering the differences among canine, molar and incisor. Modeling the above differences in swin transformer, the proposed method was expected to mine domain knowledge for more accurate caries diagnosis. To test the proposed method, a children panoramic radiograph database was built and labeled with a total of 6028 teeth. Swin transformer shows better diagnosis performance compared with typical CNN methods, which indicates the usefulness of this new technique for children caries diagnosis on panoramic radiographs. Furthermore, the proposed tooth type enhanced swin transformer outperforms the naive swin transformer with the accuracy, precision, recall, F1 and area-under-the-curve being 0.8557, 0.8832, 0.8317, 0.8567 and 0.9223, respectively. This indicates that the transformer model can be further improved with a consideration of domain knowledge instead of a copy of previous transformer models designed for natural images. Finally, we compare the proposed tooth type enhanced swin transformer with two attending doctors. The proposed method shows higher caries diagnosis accuracy for the first and second primary molars, which may assist dentists in caries diagnosis.

## 1. Introduction

Dental caries, also called tooth decay, is a common oral disease regardless of age [1]. Since a child suffers from dental caries in the primary dentition, the growth of permanent teeth will be affected [2], diagnosing dental caries in time is important [3]. To diagnose caries for children, X-ray radiography is the inspection tool most used by dentists when the teeth are difficult to diagnose by visual inspection [4,5,6]. Three classical dental X-rays (i.e., bitewing, panoramic and periapical radiograph) are commonly used, which are selected by dentists based on their needs. Recently, a panoramic radiograph was widely used for children caries diagnosis due to two benefits [7,8,9]. Firstly, a single panoramic radiograph consists of an entire dentition, which can enable a comprehensive inspection. Secondly, it provides high patient comfort for children that are not willing to cooperate.

However, performing automatic caries diagnosis based on panoramic radiographs is not easy because the bone and jaw structure around the teeth is also pictured in the panoramic radiographs. Accordingly, making caries diagnosis consists of at least two steps [10,11]: (1) extracting each tooth; and (2) performing diagnosis on each tooth. Currently, plenty of works have been proposed to automatically extract teeth from a panoramic radiograph based on algorithms such as genetic algorithms [12], image post-processing methods [13] and region CNN approaches [14,15,16,17]. However, these methods are not comparable with a nurse or a trained data annotation worker when facing various kinds of panoramic radiographs such as with metal artifact. Since most current methods focus on the second step, which requires expert dentists to make a diagnosis, the focus of the manuscript moves to step two, aiming to reduce the dentist’ workloads.

As a typical pattern recognition problem, early works conducted caries diagnosis on a tooth image using classical feature extraction and classification methods. For example, Saravanan et al. [18] used pixel intensities and spectral components as features, and found that normal and decayed teeth have very different feature ranges. Virupaiah and Sathyanarayana [19] used the Gaussian low-pass filter for features, and trained a support vector machine for classification. Recently, CNN, as a very successful deep learning method in handling 2D and 3D natural image data [20], was introduced in the field of medical image analysis, showing big performance improvements compared with conventional pattern recognition methods [21]. More and more researchers are using the state-of-the-art deep learning CNN methods for various medical fields. As for the problem of caries diagnosis in the field of stomatology, the researchers used, tested and improved the current CNN methods for automatic caries classification [9,10,22,23].

Vinayahalingam et al. [22] trained a MobileNet V2 CNN network on 400 cropped panoramic images to classify the carious lesions in mandibular and maxillary third molars, showing a high classification accuracy in caries classification in the third molars of the 100 cropped panoramic images. Bui et al. [10] tested the performances of different CNN features such as Alexnet, Googlenet, VGG16, VGG19, Resnet18, Resnet50, Resnet101 and Xception networks, which display promising results by combining with the geometric features. Haghanifar et al. [9] used several pre-trained CNN networks for feature extraction such as InceptionNet and CheNet, and adopted transfer learning and CapsNet for caries detection on panoramic radiographs. Zhou et al. [24] improved Resnet by considering information among adjacent teeth, and shows a performance improvement over the naive ResNet.

Despite the promising performance improvement of CNN methods compared to conventional machine learning approaches, researchers have found a technique called transformer, which outperforms CNN in various image analysis problems, and has quickly become mainstream in the field of computer vision [25,26]. In contrast to CNN, which uses convolution to extract high-level visual features, transformer mainly uses attention without convolution, which shows another successful image processing paradigm. More recently, an advanced transformer called swin transformer [27] won the Marr Prize, which is one of the highest honors in the field of computer vision. However, a transformer technique has not been introduced in children caries diagnosis on panoramic radiographs, which we argue could potentially improve the caries diagnosis performance.

Therefore, this study first introduces the transformer for performing children caries diagnosis on panoramic radiographs, and secondly introduces the domain knowledge of teeth to improve the transformer, which was initially designed for natural images, hoping to achieve more accurate caries diagnosis for children with primary dentition. Specifically, we use the swin transformer for caries diagnosis on a collected panoramic radiograph dataset consisting of 6028 teeth, and test its performance compared to the state-of-the-art CNN methods. Furthermore, by considering the differences among canine, molar and incisor, a tooth type enhanced swin transformer is proposed, which models different types of teeth with the shared and specific networks of a swin transformer. Compared with the naive swin transformer, the tooth type enhanced swin transformer shows a performance improvement by considering such domain-related information instead of a copy of techniques in other fields.

Finally, this paper aimed to answer three questions: if the new paradigm performs better than conventional CNN methods, can the tooth type enhanced transformer improve the caries diagnosis performance compared to the naive transformer deigned for natural images; and how does the proposed model performs compare with dentists?

## 2. Materials and Methods

### 2.1. Ethics Statement

This study was conducted with the approval of the Institutional Review Board (IRB) of Beijing Children’s Hospital, Capital Medical University, National Center for Children’s Health (IRB No.: [2022]-E-044-R). Since this work was a noninterventional study, no clinical trial was performed.

### 2.2. Materials

The panoramic radiograph database was collected in Beijing Children’s Hospital, Capital Medical University, National Center for Children’s Health from December 2015 to December 2021. The database consists of two parts: panoramic radiographs of patients who have been diagnosed with one or more caries based on their diagnostic reports, and panoramic radiographs of dental age-matched patients who are from the same hospital and have no caries according to the diagnoses of two attending doctors. All the panoramic radiographs were stored in JPEG format with a size of approximately 2441 × 1150 pixels. In summary, the database consists of 304 panoramic radiographs with a total of 6028 teeth.

**Figure 1:**
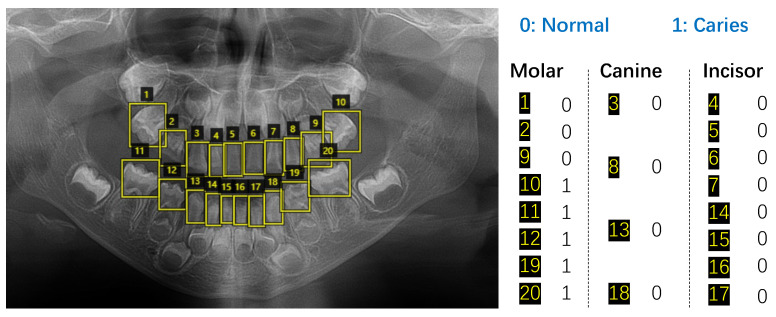
Extracting and labeling each tooth on a panoramic radiograph.

Since a panoramic radiograph has all the teeth, we extracted each tooth and annotated them with an experienced data annotation worker trained by dentists. The tool used for annotation is via [28], which is widely used in computer vision. Since different teeth are diverse in shape and size, we use different sized rectangular boxes to extract each tooth. Considering that we need to use the tooth types to improve the swin transformer, each tooth is extracted and then labeled as either a caries or not and as being either a molar, canine or incisor. An example is shown in Figure 1. In a panoramic radiograph of primary dentition, if a tooth is missing, we just ignore the tooth.

### 2.3. Methods

#### 2.3.1. Swin Transformer

Transformer is proposed to solve the poor efficiency problem of a recurrent neural network due to its sequential computing of input. It is an encoder–decoder architecture, which is based solely on self-attention and feed-forward neural network. Owing to the self-attention mechanism [29], the transformer is able to compute the representations of input in parallel, which fits the needs of modern computing devices such as GPU. Currently, transformer becomes the basic component of various foundation models such as GPT-3 [30], and has been a mainstream method in natural language processing.

The use of transformer in computer vision to analyze images has long been investigated, and it has been recognized since the introduction of vision transformer (ViT) [31] in 2020. ViT shows that, with enough training, transformer will overcome defects in the lack of inductive bias: the locality/two-dimensional neighborhood structure and translation equivariance, which are the main characteristics of CNN. Inspired from ViT, Liu et al. [27] proposed the swin transformer, which won the Marr Prize and was recognized as one of the most successful transformer models. The authors brought in a hierarchical representation computed with shifted windows, which limits the self-attention computation to non-overlapping local windows as well as allows for cross-window connection. Swin transformer is claimed to have greater efficiency with linear computational complexity with respect to image size.

In this paper, we bring in a swin transformer for children caries diagnosis on dental panoramic radiographs, which, to the best of our knowledge, is the first time this has been attempted, in the hope of providing a novel view to improve the performance of children caries diagnosis.

#### 2.3.2. Tooth Type Enhanced Swin Transformer

**Figure 2:**
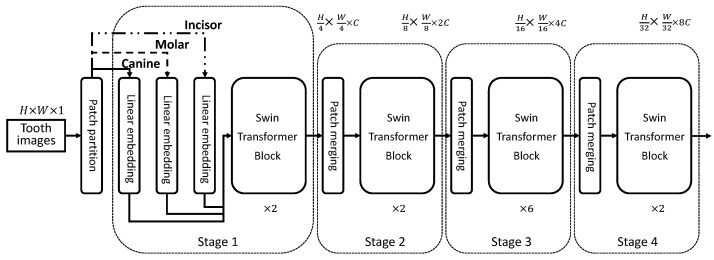
The overall framework of the proposed tooth type enhanced the swin transformer. We use different linear embeddings for different types of teeth and shared swin transformer blocks for all the teeth, which can model the tooth-specific and tooth-common characteristics, respectively. ×n means *n* successive swin transformer blocks.

The aim of the proposed tooth type enhanced swin transformer is to improve the caries classification performance, so as to help clinical applications. Considering that different types of teeth have different probabilities of developing caries, e.g., molars have a higher probability of developing caries compared to other types of teeth [32], it is natural that different teeth should use distinct classification models such as different swin transformers. On the other hand, teeth in the same mouth share the same growing environment, so we can use the shared parts of a transformer to model such common characteristics. Based on the assumption, an idea of promoting the caries classification performance is to encode above information into the swin transformer. Thus, we propose a toot-type-enhanced swin transformer (T2S-Transformer), which is shown in Figure 2.

In the T2S-Transformer, there are four stages trained end-to-end. In stage 1, each patch of a tooth image with a size of 4×4 (dimension 16) is treated as a “token” such as the ViT model. A linear embedding layer is applied on each patch to project it into an arbitrary dimension such as 96 in our experiments. The main difference between the T2S-Transformer and the naive swin transformer (S-Transformer) is the linear embeddings used, where there are three channels when a batch of input tooth images consisting of canine, molar and incisor.

(1) $$x={\mathrm{LN}}_{k}\left(x_{k}\right)$$

where $x_{k}$ is the feature of the canine, molar or incisor, and LN is the linear embedding. Based on such a design, tooth-specific characteristics can be modeled.

**Figure 3:**
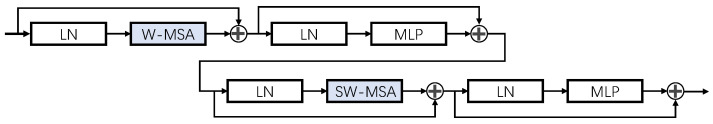
Blocks of two successive swin transformer blocks that use multi-head self-attention (MSA) modules with regular (W-MSA) and shifted windowing (SW-MSA) configurations. More details can refer to [27].

For the swin transformer blocks used in each of the four stages, they share the same parameters between different types of teeth, which models the tooth-common characteristics. The specific architecture of two successive swin transformer blocks is shown in Figure 3, and it is calculated as:

(2) $${\tilde{z}}^{l}=\text{W-MSA}\left(\mathrm{LN}\left(z^{l-1}\right)\right)+z^{l-1}$$

(3) $$z^{l}=\mathrm{MLP}\left(\mathrm{LN}\left({\tilde{z}}^{l}\right)\right)+{\tilde{z}}^{l}$$

(4) $${\tilde{z}}^{l+1}=\text{SW-MSA}\left(\mathrm{LN}\left(z^{l}\right)\right)+z^{l}$$

(5) $$z^{l+1}=\mathrm{MLP}(\mathrm{LN}\left({\tilde{z}}^{l+1}\right))+{\tilde{z}}^{l+1}$$

where ${\tilde{z}}^{l}$ and ${\tilde{z}}^{l+1}$ are the outputs of W-MSA and SW-MSA, respectively, $z^{l}$ and $z^{l+1}$ are the outputs of the *l*-th and (l+1)-th layers, respectively, and $z^{0}=x$. MLP is a two-layer multilayer perceptron. W-MSA and SW-MSA are the regular and shifted windowing multi-head self attention modules based on the patch-merging module [27].

Based on the output of stage 4, we can obtain the final representation of a tooth image as:

(6) $$z^{\mathrm{final}}=\mathrm{avgpool}\left(z^{L}\right)$$

where avgpool is the average pooling of all the patches in *L* layers. With the final representation, we use a softmax-activated classifier layer for caries classification, and a cross-entropy loss is used for model training.

### 2.4. Model Training

In Section 2.2, a tooth image database is collected consisting of 6028 teeth, which will be divided into training, validation and testing sets. We use the training set to train all the models, save the model according to the performance on the validation set, and test the models on the testing set with the saved model. Before training, we resize all the images of size 224×224, which is essential because different tooth images have different sizes due to the non-restricting rectangular boxes. Additionally, we perform image contrast enhancement by adjusting the intensity of each pixel, which we found to be useful in the experiments.

As for the hyper-parameters used, we set the dimension (*C* in Figure 2), mini-batch size, learning rate and the maximum training iterations to be 96, 32, $10^{-3}$ and 3000, respectively. As for the computing resources, we used an assembled server, which is configured with 2 × Intel(R) Xeon(R) Gold 6240R CPU and NVIDIA RTX 2080 Ti GPU (12 GB Ram).

### 2.5. Performance Evaluation

Five typical classification metrics are used for the performance evaluation such as in previous studies, which are accuracy, precision, recall, F1-score and area-under-the-curve (AUC). To calculate the metrics, we suppose that *TP*, *FP*, *FN* and *TN* are true positive, false positive, false negative and true negative, respectively, and then we can directly obtain the metrics as:

(7) $$\mathrm{Accuracy}=\frac{TP+TN}{TP+TN+FP+FN}$$

(8) $$\mathrm{Precision}=\frac{TP}{TP+FP}$$

(9) $$\mathrm{Recall}=\frac{TP}{TP+FN}$$

(10) $$F1=\frac{2\times \mathrm{Precision}\times \mathrm{Recall}}{\mathrm{Precision}+\mathrm{Recall}}$$

To obtain the AUC, we first plot the receiver operating characteristic (ROC) curve, and then calculate the area under ROC as AUC. For all the metrics, a higher value means a better performance.

We made a comparison thanks to the two dentists, and to measure the agreement between the observations of the two dentists, a kappa coefficient [33] was used and calculated as:

(11) $$\kappa =\frac{P_{o}-P_{e}}{1-P_{e}}$$

where $P_{o}$ and $P_{e}$ are the percent agreement and the expected percent agreement, respectively. The division corrects for agreement due to chance. Usually, 0.8–1.0 of kappa value indicates an almost perfect agreement, and 0.6–0.8 represents a substantial agreement.

## 3. Results

### 3.1. Dataset

In total, we have 6028 teeth, comprising 3039 and 2989 teeth with and without caries, respectively. To perform caries diagnosis, we split the teeth into training, validation and testing sets. The training set consists of 244 panoramic radiographs with 4833 teeth, among which there are 2432 and 2401 teeth with and without caries, respectively. The validation set consists of 30 panoramic radiographs with 599 teeth, among which there are 320 and 279 teeth with and without caries, respectively. The testing set consists of 30 panoramic radiographs with 596 teeth, among which there are 287 and 309 teeth with and without caries, respectively.

### 3.2. Compared to Typical CNN Methods

We select four typical CNN methods as baseline to verify the swin transformer for children caries diagnosis. The four CNN methods are AlexNet, GoogleNet, SeNet and ResNet [16], and the comparison result is shown in Table 1. It can be seen that the swin transformer outperforms all the strong CNN baselines, which shows the potential of the new paradigm on the problem of children caries diagnosis on panoramic radiographs.

### 3.3. Performance of the Proposed T2S-Transformer

Based on swin transformer, we propose a tooth type enhanced swin transformer for further improving the caries classification performance by considering that different types of teeth have different probabilities of being caries. Table 2 details the caries classification performance of the proposed T2S-Transformer and the naive S-Transformer.

**Figure 4:**
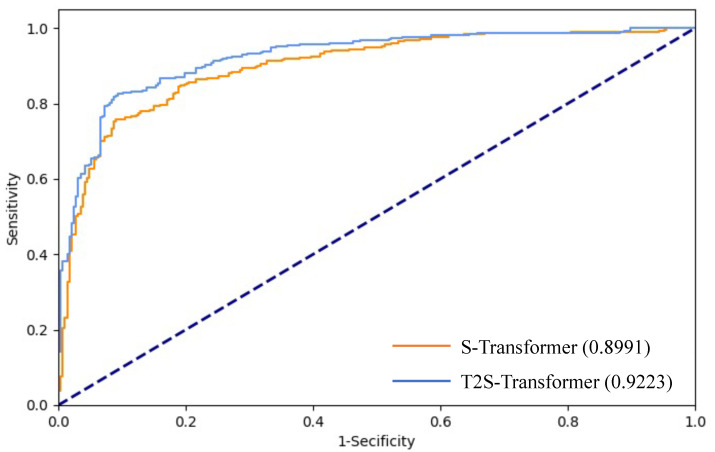
Receiver operating characteristic (ROC) curves of the proposed T2S-Transformer and the naive S-Transformer. Numbers in parentheses show the AUC values of the two methods.

Due to a consideration of the domain knowledge, the proposed T2S-Transformer outperforms the naive transformer by about 3% in terms of accuracy, precision, recall, F1 and AUC. Figure 4 shows the ROC curves of T2S-Transformer and S-Transformer, which further validates the helpfulness of using domain knowledge for caries diagnosis.

**Figure 5:**
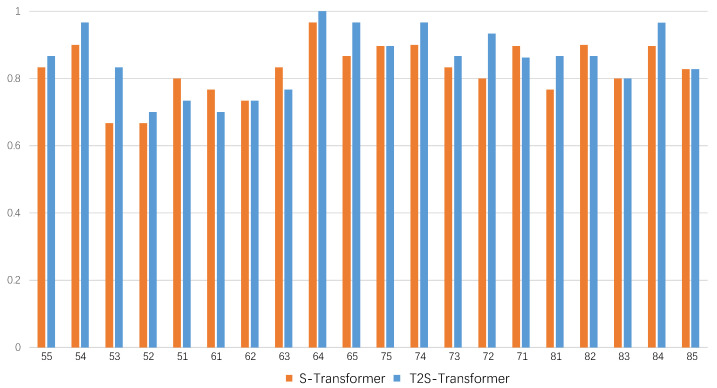
Classification accuracy of each tooth for the proposed T2S-Transformer and the naive S-Transformer. Numbers in the horizontal ordinate denote the tooth positions.

For the performance in terms of each tooth, we plot the accuracy compared to the naive S-Transformer, as shown in Figure 5. It can be seen that the proposed method outperforms the naive S-Transformer in most tooth positions. The improvements in the performance of the naive S-Transformer for teeth 72, 81 and 53 were larger than 10%.

### 3.4. Comparison with Dentists

To further validate the proposed method, we made a comparison with different dentists. The diagnosis performances of the two attending doctors (AD) and the proposed T2S-Transformer are presented in Table 3. It can be seen that the caries diagnosis performance of the proposed T2S-Transformer is a bit worse than a doctor’s diagnosis, which indicates that the deep learning methods must be further improved in the future. However, the time used for a panoramic radiograph image diagnosis is much shorter compared to going to the dentists, which shows the advantage of the deep learning models.

**Figure 6:**
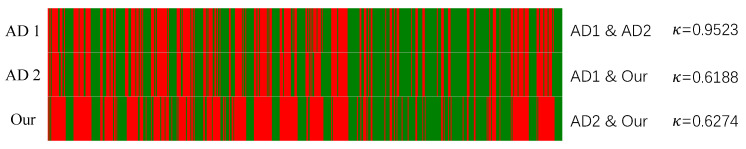
Observations by the dentists and predicted results of our method. Red and green lines represent caries and normal teeth, respectively. The kappa values are reported.

To measure the agreement between the observations of the two doctors, we plot the observations along with the kappa value, as shown in Figure 6. It can be seen that the kappa value of the two attending doctors is 0.9523, indicating almost perfect agreement. The kappa values of the proposed method and each dentist are also calculated (0.6188, 0.6274), showing substantial agreement.

Table 4 further shows the diagnosis performance of each tooth position of the proposed T2S-Transformer compared to the attending doctors. From the results, we can see a better accuracy obtained on the primary molars, i.e., tooth positions 54, 55, 64, 65, 74, 75, 84 and 85. This shows a shining point of the proposed method, which displays the attending doctors’ level in caries diagnosis of the primary molars on panoramic radiographs.

## 4. Discussion

CNN, as a very successful deep learning technique for performing natural image analysis, is currently being introduced in medical image analysis, and promising results have been achieved compared to conventional pattern recognition methods. Benefiting from the interdisciplinary study between deep leaning and stomatology, studies have brought some state-of-the-art CNN methods for caries diagnosis. For example, Vinayahalingam et al. [22] trained a MobileNet V2 CNN for mandibular and maxillary third-molar caries classification. Bui et al. [10] used various CNN methods for feature extraction, including Alexnet, Googlenet, VGG16, VGG19, Resnet18, Resnet50, Resnet101 and Xception networks. Zhou et al. [24] proposed context-aware ResNet for caries diagnosis by considering information among adjacent teeth.

Despite the promising performance improvement of CNN methods in caries diagnosis, the transformer is showing potential for natural image analysis, and currently becomes mainstream in the field of computer vision. It is a very different deep learning framework compared to CNN. In this study, we aim to introduce the transformer for more accurate children caries diagnosis based on dental panoramic radiographs. We tested the performance of the swin transformer, one of the best transformer models, and compared it to several classical CNN methods such as AlexNet, GoogleNet, SeNet and ResNet. The results show the potential of this new paradigm regarding children caries diagnosis on panoramic radiographs. The performance improvements were of at least 2% in terms of accuracy, precision, F1 and AUC, and of approximately 5% in terms of accuracy and precision.

In this study, we also aim to promote conventional transformer models for more accurate children caries classification, hoping to help clinical applications. By considering the fact that different types of teeth have different probabilities of being caries, and embedding such domain knowledge into transformer networks, the proposed method can better model the characteristics of dental panoramic radiographs that are different from natural images. A tooth type enhanced swin transformer is proposed by modeling tooth-specific and tooth-common characteristics. Since only several linear embeddings are added to revise the naive swin transformer, a small number of parameters are added, making no increment in computational burden.

As for the performance, the proposed T2S-Transformer outperforms the naive S-Transformer by approximately 3% in terms of accuracy, precision, recall, F1 and AUC. This validates the helpfulness of using domain knowledge for children caries diagnosis. We consider the performance comparison for each tooth, the improvements for tooth positions 72, 81 and 53 are more than 10%. We also plot the receiver operating characteristic curves for the proposed T2S-Transformer and the naive S-Transformer, showing a more intuitive advantage.

This study further validates the proposed method by making a comparison with two attending doctors. Almost all the metrics of the proposed T2S-Transformer showed a slightly worse performance than the professional attending doctors, except for the precision metric. This indicates the potential of our model, but also shows a further study required to reach professional dentists in an all-round level. On the other hand, the diagnosis speed of the proposed T2S-Transformer is significantly faster than that of the attending doctors, which is less than 1 s compared to more than 1 min for doctors. Accordingly, introducing the proposed model for assisting caries diagnosis will significantly shorten the diagnosis time for dentists, especially for those with little experience.

This study further conducted experiments to show the diagnosis performance of each tooth position of the proposed T2S-Transformer compared to the attending doctors. An encouraging result was obtained when the proposed model performed better than the doctors in terms of accuracy for tooth positions 54, 55, 64, 65, 74, 75, 84 and 85. This indicates that T2S-Transformer reaches the level of the attending doctors in the children caries diagnosis of the primary molars. This observation seems obvious because panoramic radiographs are not reliable in the assessment of heart tissues in the anterior section of the maxilla and mandible due to the blurring of the imaged structures. However, the proposed method provides a potential way for caries diagnosis for primary molars when only panoramic radiographs can be provided. Considering that the proposed model also has the diagnosis speed advantage, it may be deployed to help dentists perform caries diagnosis in hospital.

This study also has some limitations required for further study. Firstly, the proposed T2S-Transformer focuses on caries diagnosis based on each tooth extracted from the dental panoramic radiographs. However, the tooth extraction stage was ignored, and it will limit the clinical use due to extra human annotations. Secondly, we regard children caries diagnosis as a two-class problem, which is basic but may not be enough to assist dentists in making a more accurate diagnosis of what degree the caries is. Thirdly, we are at a stage where artificial intelligence techniques are being trained for caries diagnosis on X-rays. However, as orthopanormics X-ray is not a diagnostic investigation of high sensitivity for caries diagnosis [34,35], it would be appropriate to introduce other goals for the use of artificial intelligence in dentistry.

## 5. Conclusions

By introducing the transformer for children caries diagnosis, we show its advantages compared to classical CNN methods, validating the potential of bringing such a new deep learning paradigm into the field of children caries diagnosis on panoramic radiographs. Furthermore, a tooth type enhanced swin transformer was proposed, showing an improvement in caries diagnosis performance. This validates the usefulness of considering the domain knowledge compared to a copy of previous models designed for natural images. Finally, the caries diagnosis performance reached the level of the attending doctor for primary molars, indicating a the potential for helping dentists in the clinical performance of caries diagnosis.

## Figures and Tables

**Table 1:** Performance comparison between the swin transformer and the typical CNN baselines. We follow the authors’ instructions on implementing CNN methods, and report their best performance.

Methods	Accuracy	Precision	Recall	F1	AUC
AlexNet	0.6040	0.6181	0.6181	0.6181	0.6547
GoogleNet	0.6376	0.6317	0.7217	0.6737	0.6633
SeNet	0.7836	0.8000	0.7767	0.7882	0.8520
ResNet	0.7768	0.8056	0.8049	0.8052	0.8490
S-Transformer	0.8272	0.8576	0.7994	0.8275	0.8991

**Table 2:** Performance comparison between the proposed T2S-Transformer and the naive S-Transformer.

Methods	Accuracy	Precision	Recall	F1	AUC
S-Transformer	0.8272	0.8576	0.7994	0.8275	0.8991
T2S-Transformer	0.8557	0.8832	0.8317	0.8567	0.9223

**Table 3:** Performance comparison between the proposed T2S-Transformer and the two attending doctors (average and individual performances are reported).

Methods	Accuracy	Precision	Recall	F1	Time (s)
T2S-Transformer	0.8557	0.8832	0.8317	0.8567	0.6897
AD	0.8842 (0.8808, 0.8876)	0.8509 (0.8473, 0.8545)	0.9417 (0.9365, 0.9469)	0.8940 (0.8897, 0.8983)	64.5000 (69.0000, 60.0000)

**Table 4:** Classification accuracy of each tooth for the proposed T2S-Transformer and two attending doctors (AD, average performance is reported).

Position	55	54	53	52	51
T2S-Transformer	0.8667	0.9667	0.8333	0.7000	0.7333
AD	0.7667	0.9000	0.9333	0.9333	0.9333
**Position**	**61**	**62**	**63**	**64**	**65**
T2S-Transformer	0.7000	0.7333	0.7667	1.0000	0.9667
AD	0.8333	0.9333	0.8667	0.8667	0.8333
**Position**	**75**	**74**	**73**	**72**	**71**
T2S-Transformer	0.8966	0.9667	0.8667	0.9333	0.8621
AD	0.8276	0.9000	0.8333	1.0000	0.9310
**Position**	**81**	**82**	**83**	**84**	**85**
T2S-Transformer	0.8667	0.8667	0.8000	0.9655	0.8276
AD	0.9333	0.9000	0.9000	0.9310	0.7241

## Data Availability

Data used in this study were obtained from Department of Stomatology, Beijing Children’s Hospital, Capital Medical University, National Center for Children’s Health, and researchers can apply for use (through the corresponding author) with the permission of the Institutional Review Board (IRB) of Beijing Children’s Hospital, Capital Medical University, National Center for Children’s Health.
